# Peculiar Ca^2+^ Homeostasis, ER Stress, Autophagy, and TG2 Modulation in Celiac Disease Patient-Derived Cells

**DOI:** 10.3390/ijms24021495

**Published:** 2023-01-12

**Authors:** Silvia Sposito, Agnese Secondo, Antonio Massimiliano Romanelli, Antonio Montefusco, Merlin Nanayakkara, Salvatore Auricchio, Maria Vittoria Barone, Ivana Caputo, Gaetana Paolella

**Affiliations:** 1 Department of Chemistry and Biology, University of Salerno, 84084 Fisciano, Italy; 2 Division of Pharmacology, Department of Neuroscience, Reproductive and Odontostomatological Sciences, School of Medicine, University Federico II, 80138 Naples, Italy; 3 Department of Translational Medical Science, University Federico II, 80138 Naples, Italy; 4 European Laboratory for the Investigation of Food-Induced Diseases (ELFID), University Federico II, 80138 Naples, Italy

**Keywords:** celiac disease, Ca^2+^ homeostasis, type 2 transglutaminase, unfolded protein response, ER stress, autophagy, thapsigargin

## Abstract

Celiac disease (CD) is an inflammatory intestinal disease caused by the ingestion of gluten-containing cereals by genetically predisposed individuals. Constitutive differences between cells from CD patients and control subjects, including levels of protein phosphorylation, alterations of vesicular trafficking, and regulation of type 2 transglutaminase (TG2), have been reported. In the present work, we investigated how skin-derived fibroblasts from CD and control subjects responded to thapsigargin, an endoplasmic reticulum ER stress inducer, in an attempt to contribute to the comprehension of molecular features of the CD cellular phenotype. We analyzed Ca^2+^ levels by single-cell video-imaging and TG2 activity by a microplate assay. Western blots and PCR analyses were employed to monitor TG2 levels and markers of ER stress and autophagy. We found that the cytosolic and ER Ca^2+^ level of CD cells was lower than in control cells. Treatments with thapsigargin differently activated TG2 in control and CD cells, as well as caused slightly different responses regarding the activation of ER stress and the expression of autophagic markers. On the whole, our findings identified further molecular features of the celiac cellular phenotype and highlighted that CD cells appeared less capable of adapting to a stress condition and responding in a physiological way.

## 1. Introduction

Celiac disease (CD) is a widely diffused inflammatory enteropathy of the small intestine affecting genetically-predisposed individuals exposed to wheat gluten through the diet [1,2]. Gluten comprises two fractions, gliadin and glutenin, depending on their water solubility, characterized by a high content of Pro and Gln residues [3]. For this reason, gluten is only partially digested in the gut lumen. Several peptides are recognized in the lamina propria by antigen-presenting cells which possess particular human leukocyte antigens (HLA)-II haplotypes, typically DQ2 or DQ8, thus triggering an adaptive immune response against gluten [1]. As a consequence, gut inflammation occurs, causing an extended mucosal remodeling with atrophy, hyperplasia, and lymphocytic infiltration [2]. In relation with this mucosal damage, gastrointestinal CD manifestations are very common [4]. The interaction between HLA-DQ2/8 and gluten peptides becomes stronger when peptides are enzymatically modified by type 2 tranglutaminase (TG2) [5]), a ubiquitous Ca^2+^-dependent enzyme whose intestinal expression is increased by inflammatory stimuli. TG2 is a transamidase that normally forms isopeptide bonds between the γ-carboxamide group of a Gln residue and an ε-amino group of a Lys residue belonging to the same protein or to different proteins [6]. Other primary aminic groups can also be used by TG2. In rare environmental conditions, i.e., slightly acidic pH and a virtual absence of aminic groups, TG2 can deamidate the γ-carboxamide group of a specific Gln, forming a Glu residue [6]. This reaction plays a crucial role in the loss of tolerance to gluten, as the net negative charge presented by the Glu residue renders the gluten peptide more affine to the DQ2/8 groove, thus potentiating antigen presentation to the immune system [5,7]. In addition, TG2 is able to form cross-links between Gln residues belonging to gluten peptides and self-Lys residues. According to a hapten-carrier-like model, complexes between gluten and TG2 trigger an autoimmune response to TG2 itself [8].

In skin-derived fibroblasts from CD and control biopsies, we recently demonstrated a different response, in terms of TG2 expression and activation, in the presence of the α-gliadin peptide 31–43 (P31-43) [9], the main peptide responsible for the innate immune response in CD [10,11]. Since P31-43 was able to mobilize Ca^2+^ ions from intracellular deposits, cytosolic TG2 was activated by the exposition to P31-43 [12]. However, reduced activation in celiac fibroblasts was observed; thus, we hypothesized that Ca^2+^ ions may regulate each cellular function differently depending on the route and the signaling pathway triggered.

In the present work, we aimed to gain more insights about Ca^2+^ homeostasis in CD and control cells and also to investigate two biological processes which are related to Ca^2+^ mobilization, i.e., endoplasmic reticulum (ER) stress and autophagy, also focusing on TG2 modulation. Thapsigargin (THP), a known inducer of ER stress and autophagy, has been employed here to discriminate different responses in celiac and control skin-derived fibroblasts. We found that celiac cells had lower levels of Ca^2+^ ions in the cytosol and in the ER lumen, with consequent lower TG2 activation, with respect to the control cells. In addition, CD cells displayed a more intense unfolded protein response (UPR) to THP with respect to the control fibroblasts, and also presented defective autophagy.

## 2. Results

### 2.1. Effect of THP on Fibroblasts’ Viability

**Figure 1:**
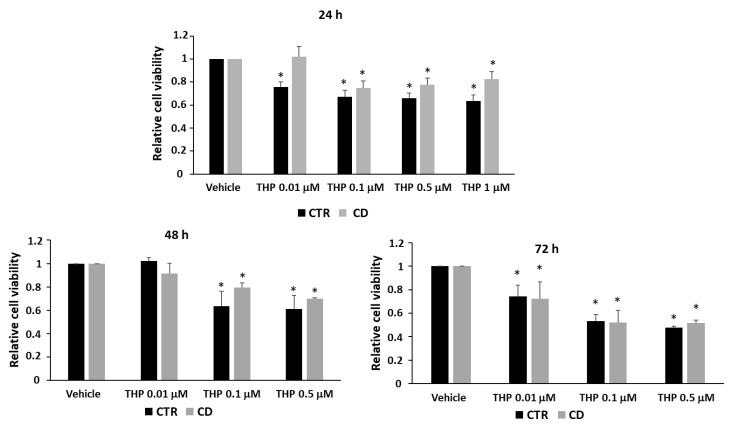
Relative cell viability measured by an MTT assay after treatments with THP ranging from 0.01 to 1 µM (for 24 h) or from 0.01 to 0.5 µM (for 48 and 72 h). Basal viability is measured in the presence of the vehicle (DMSO 0.05%). In all experiments, the vehicle reduced cell viability by no more than 10–15%. Data are reported as means ± standard error (SE) from two independent experiments, each performed in triplicate, on three non-CD cultures and three CD cultures. * *p* < 0.05 versus the respective vehicle.

We first studied to what extent THP affected the cell viability of primary fibroblasts by an MTT assay. We found that, after 24 h, THP reduced cell viability both in CD and in control cells; however, control cultures appeared more sensible than the celiac ones (Figure 1). Indeed, at the lowest tested concentration (0.01 µM), THP reduced the viability of control cells by about 20%, whereas CD fibroblasts were unaffected. After 48 h, we still observed a slightly greater sensitivity to THP of control cells with respect to the CD ones, whereas after 72 h of treatments, control and CD cells responded in a similar way (Figure 1).

### 2.2. Different Basal Ca^2+^ Levels in Control and CD Fibroblasts

**Figure 2:**
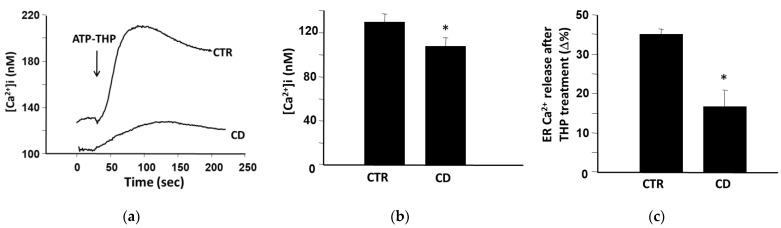
Ca^2+^ level measurement in skin-derived fibroblasts. (**a**) Superimposed single-cell traces representative of the rapid effect of THP on [Ca^2+^]i in one representative non-CD sample and one representative CD sample. Starting time of perfusion with THP is indicated by the arrow. (**b**) Quantification of basal [Ca^2+^]i in four non-CD and three CD cultures. (**c**) Quantification of Ca^2+^ release from ER after treatment with THP in four non-CD and three CD cultures. For each experiment, 40–50 individual cells were monitored. * *p* < 0.05 versus controls (Student’s *t*-test for unpaired data).

We studied differences regarding the intracellular Ca^2+^ homeostasis in CD and control cells, particularly regarding the ER, which is also a target of the Ca^2+^-mobilizing activity of P31-43 [12]. To this aim, we performed single-cell Fura-2 acetoxymethyl ester (Fura-2AM) microfluorimetric measurements. We found that the basal intracellular [Ca^2+^] was significantly lower in fibroblasts of CD subjects than in cells from control cultures (Figure 2a,b). In addition, the perfusion of THP in a natural Krebs medium produced a rapid increase of cytosolic Ca^2+^ concentration both in CD and non-CD cells, but Ca^2+^ ions released from ER were significantly less in CD cells than in control ones (Figure 2c).

### 2.3. Differences in THP-Induced ER Stress in Control and CD Fibroblasts

**Figure 3:**
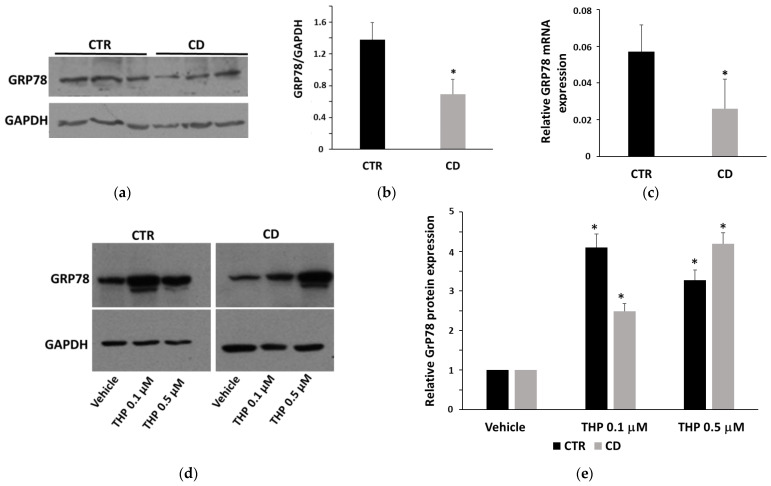

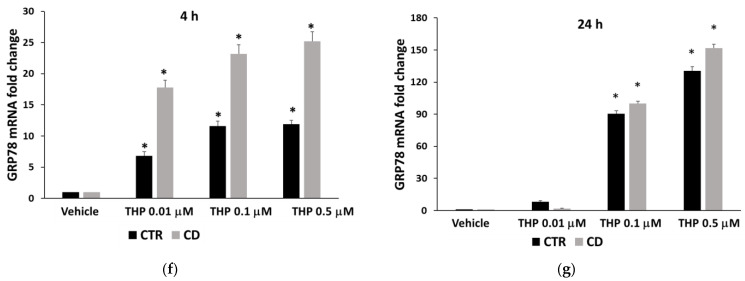
GRP78 expression. (**a**) Representative Western blot of the basal level of GRP78 in samples from three control and three celiac cultures. (**b**) Densitometric analyses of GRP78 basal levels, normalized with respect to GAPDH expression, relative to five controls and five celiac cultures. (**c**) Relative basal GRP78 mRNA levels measured in cultures from three control and three CD subjects. In panels (**a**–**c**), data are reported as mean ± SE. * *p* < 0.05 vs. controls (Student’s *t*-test for unpaired data). (**d**) Representative Western blot anti-GRP78 on samples from one control and one celiac culture in the presence of THP for 24 h. (**e**) Densitometric analyses relative to Western blot anti-GRP78 on samples from three control and three celiac cultures in the presence of THP. (**f**,**g**) Relative GRP78 mRNA levels measured in samples from three control and three celiac cultures in the presence of THP for 4 h and for 24 h, respectively. In (**e**–**g**) protein and mRNA levels are normalized with respect to GAPDH expression and expressed as variation with respect to the relative vehicle-treated sample. Data are reported as mean ± SE. * *p* < 0.05 vs. the respective vehicle.

**Figure 4:**
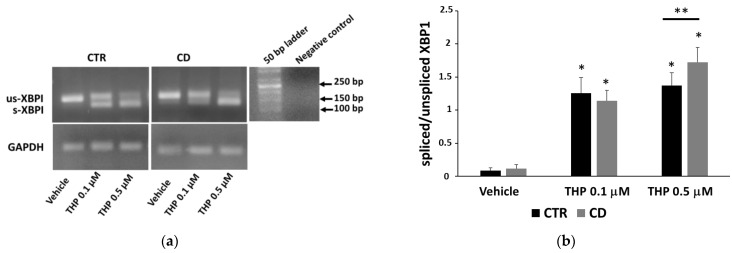
Detection of XBP1 splicing after treatments of control and CD cells with THP for 4 h. (**a**) Spliced (s) and unspliced (us) forms of XBP1 in representative samples are detected in a 2.5% agarose gel after staining with ethidium bromide. (**b**) Quantification of XBP1 splicing in cultures from three control and three CD subjects expressed as a ratio between densitometric values of s-XBP1 and us-XBP1. Data are reported as mean ± SE. * *p* < 0.05 vs. the respective vehicle. ** *p* < 0.05 as indicated (Student’s *t*-test for unpaired data).

Based on differences found in the regulation of intracellular Ca^2+^ homeostasis regarding ER, we investigated the occurrence of UPR in THP-treated cells by analyzing the expression of the biochemical marker 78-kDa Glucose-Regulated Protein (GRP78). Western blot and real-time PCR analyses revealed that basal GRP78 in CD cells was invariably less expressed than in control cells (Figure 3a–c). After 24 h of treatment with THP at concentrations of 0.1 and 0.5 μM, we observed a slightly different response in CD and non-CD cells. In control cells, GRP78 already strongly increased at 0.1 µM; in CD cells, GRP78 increment was less evident, but the response at 0.5 µM was very intense (Figure 3d,e). Real-time PCR analyses on samples treated with THP for 4 h revealed that GRP78 mRNA increased more in CD cells than in control ones (Figure 3f). However, after 24 h of treatment, mRNA fold-increase was equivalent in both groups of cells (Figure 3g). As a second marker of UPR, we monitored the occurrence of X-Box Binding Protein 1 (XBP1) splicing. Analyses by conventional semi-quantitative PCR highlighted that in both groups of cells, an increase of spliced XBP1 after treatments with THP for 4 h was evident; however, in control cells, we observed a strong and similar response at 0.1 and 0.5 µM of THP (Figure 4), probably indicating that, after a rapid response, cells tend to adapt to the stress. In CD cells, splicing was already evident at 0.1 µM of THP, but it became still more intense at 0.5 µM of THP (Figure 4).

### 2.4. Differences in THP-Induced Autophagy in Control and CD Fibroblasts

**Figure 5:**
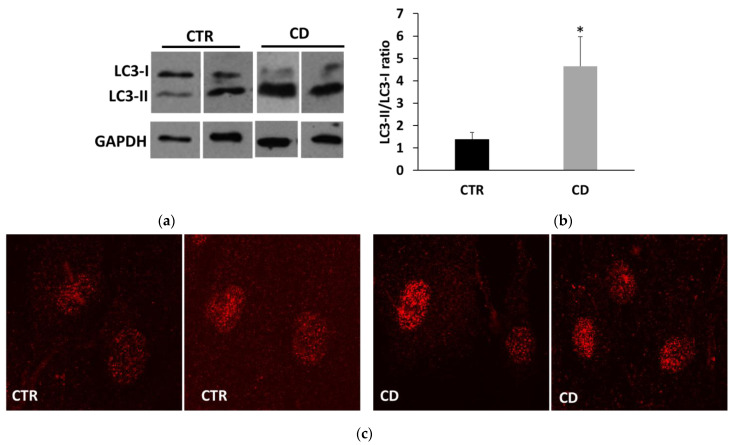
Basal LC3 expression. (**a**) Representative Western blot anti-LC3 showing basal levels of LC3-I and LC3-II on samples from two control and two celiac cultures. (**b**) Representation of ratio between LC3-II and LC3-I forms in samples from four controls and four CD cultures. Data are reported as mean ± SE. * *p* < 0.05 versus controls (Student’s *t*-test for unpaired data). (**c**) Representative confocal images of LC3 in cells from two control cultures and two CD ones (63× magnification).

**Figure 6:**
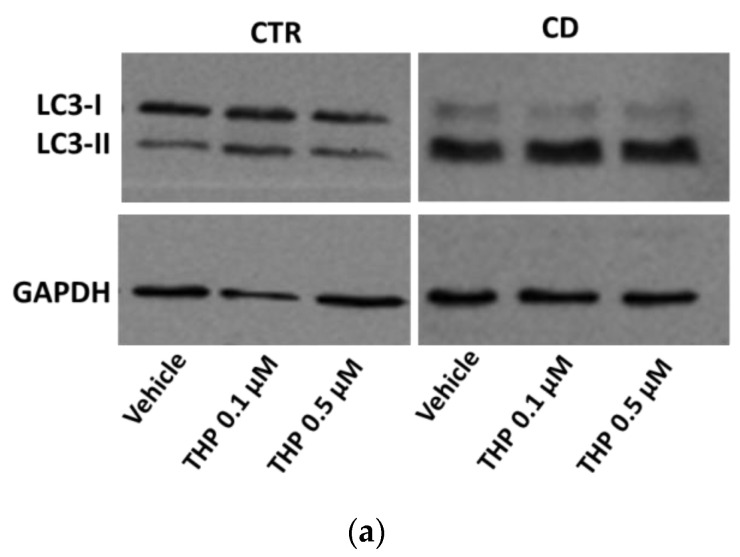

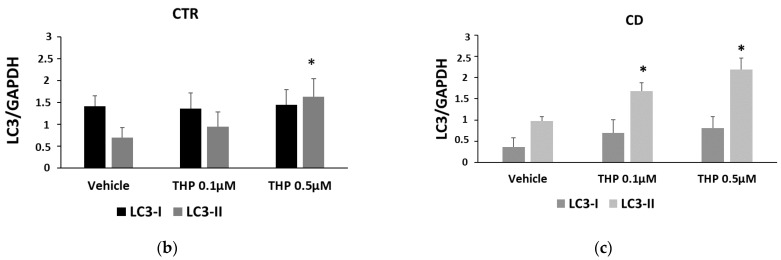
LC3 expression in cells treated with THP (**a**) Representative Western blot anti-LC3 on samples from one control and one celiac culture in the presence of THP for 24 h. (**b**,**c**) Densitometric analyses relative to Western blot anti-LC3 on samples from three control and three celiac cultures, respectively, in the presence of THP. Protein levels of LC3-I and LC3-II are normalized with respect to GAPDH expression. Data are reported as mean ± SE. * *p* < 0.05 vs. the respective vehicle.

**Figure 7:**
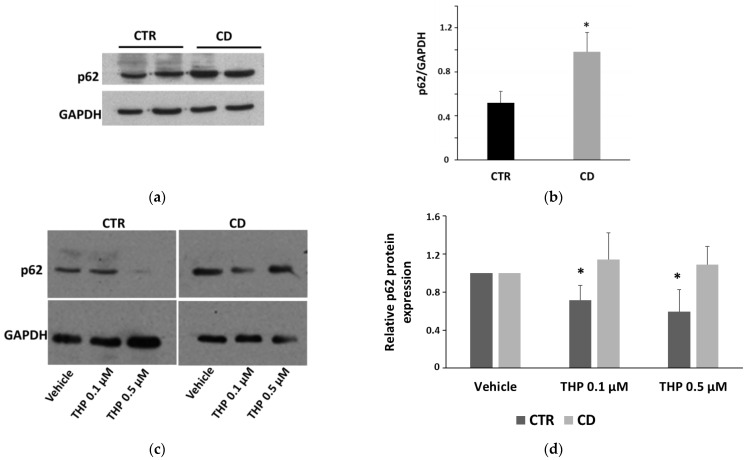
p62 expression. (**a**) Representative Western blot of basal level of p62 in samples from two control and two celiac cultures. (**b**) Densitometric analyses of p62 basal levels, normalized respect to GAPDH expression, relative to samples from four controls and from four celiac cultures. Data are reported as mean ± SE. * *p* < 0.05 vs. controls (Student’s *t*-test for unpaired data). (**c**) Representative Western blot anti-p62 on samples from one control and one celiac culture in the presence of THP for 24 h. (**d**) Densitometric analyses relative to Western blot anti-p62 on samples from three control and three celiac cultures in the presence of THP. Protein levels are normalized with respect to GAPDH expression and reported as variations with respect to the relative vehicle-treated sample. Data are reported as mean ± SE. * *p* < 0.05 vs. the respective vehicle.

**Figure 8:**
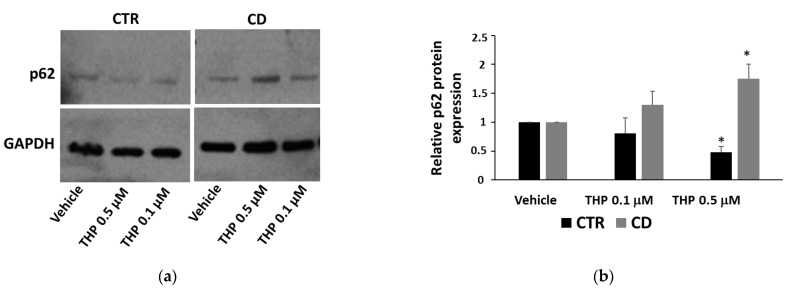
p62 expression in fibroblasts treated for 4 with THP. (**a**) Representative Western blot anti-p62 on samples from one control and one celiac culture. (**b**) Densitometric analyses of p62 basal levels relative to samples from three controls and from three celiac cultures in the presence of THP. Protein levels are normalized with respect to GAPDH expression and reported as variations with respect to the relative vehicle-treated sample. Data are reported as mean ± SE. * *p* < 0.05 vs. the respective vehicle.

Since ER stress is a potent trigger of autophagy, we investigated whether THP treatments produced differential autophagic responses in CD and non-CD fibroblasts. Changes in the level of the two forms of LC3, LC3-I, and LC3-II, were monitored. We first observed that the ratio between basal LC3-II and LC3-I was generally higher in CD cells than in control ones (Figure 5a,b). Moreover, confocal images of basal LC3 showed a more marked presence of large perinuclear vesicles in CD samples than in control ones (Figure 5c). The treatment for 24 h with THP caused a slight but significant increase of LC3-II in both groups, indicating an accumulation of autophagosomes (Figure 6). Then we analyzed another marker of autophagy, p62, indicating the progression along the autophagic pathway. We found that the basal p62 level was constitutively higher in CD cells than in non-CD cells (Figure 7a,b). In the presence of THP, we appreciated a reduction of p62 level only in control cells treated for 24 h but not in CD samples (Figure 7c,d). This difference was even more evident at a shorter time of treatment with THP (4 h); in this case, the effect of p62 degradation during autophagy was evident in control cells, whereas, in CD fibroblasts, p62 level clearly increased (Figure 8).

**Figure 9:**
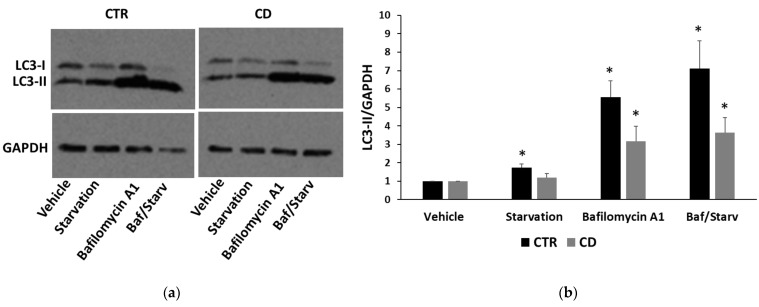

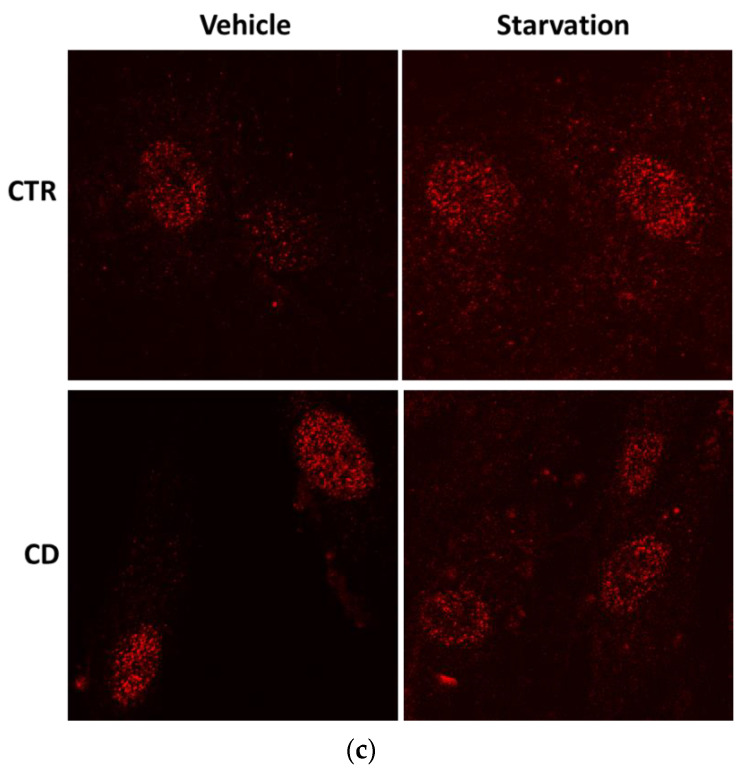
LC3 levels in the presence of starvation medium and/or of bafilomycin A1. (**a**) Representative Western blot anti-LC3 on samples from one control and one celiac culture, treated with bafilomycin A1 1 µM for 4 h; where indicated, cells were previously starved for 2 h. (**b**) Densitometric analyses of LC3-II isoform levels relative to samples from three controls and three celiac cultures treated as in (**a**). Protein levels are normalized with respect to GAPDH expression and reported as variations with respect to the relative vehicle-treated sample. Data are reported as mean ± SE. * *p* < 0.05 vs. the respective vehicle. (**c**) Confocal immunofluorescence images of fibroblasts from control and celiac subjects stained with anti-LC3 antibodies (magnification 63×).

Altogether, these data suggested that autophagic flux could be defective in CD cells. To verify this suggestion, we tested the effect of starvation, a stimulus of autophagy, and of bafilomycin A1, an inhibitor of the autophagic flux. In control fibroblasts, we observed that starvation caused a slight but significant increase of the LC3-II isoform and an accumulation of LC3-positive autophagosomes (Figure 9a–c). Bafilomycin A1 alone strongly increased the LC3-II form, and this increase was more evident (with the parallel reduction of the LC3-I form) when cells were previously starved (Figure 9a,b). This behavior indicated that autophagic flux was really enhanced. In CD samples, the effect of starvation was poorly evident, as shown by the level of LC3-II isoform in Western blot (Figure 9a,b) and by the immunofluorescent staining of LC3-positive vesicles (Figure 9c). Bafilomycin A1 alone caused a consistent accumulation of LC3-II, but the effect, together with the starvation, was less evident with respect to control samples (Figure 9a,b). Data indicated that in CD cells, autophagy could be partially engulfed.

### 2.5. TG2 Activity and Expression in Response to THP

**Figure 10:**
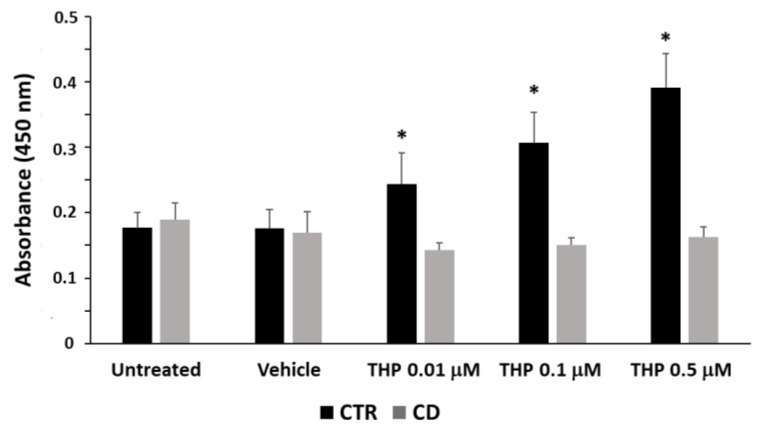
Enzymatic activity of intracellular TG2 expressed as absorbance at 450 nm in skin-derived fibroblasts from three controls and three CD subjects. Data are reported as mean ± SE. * *p* < 0.05 versus respective untreated samples.

**Figure 11:**
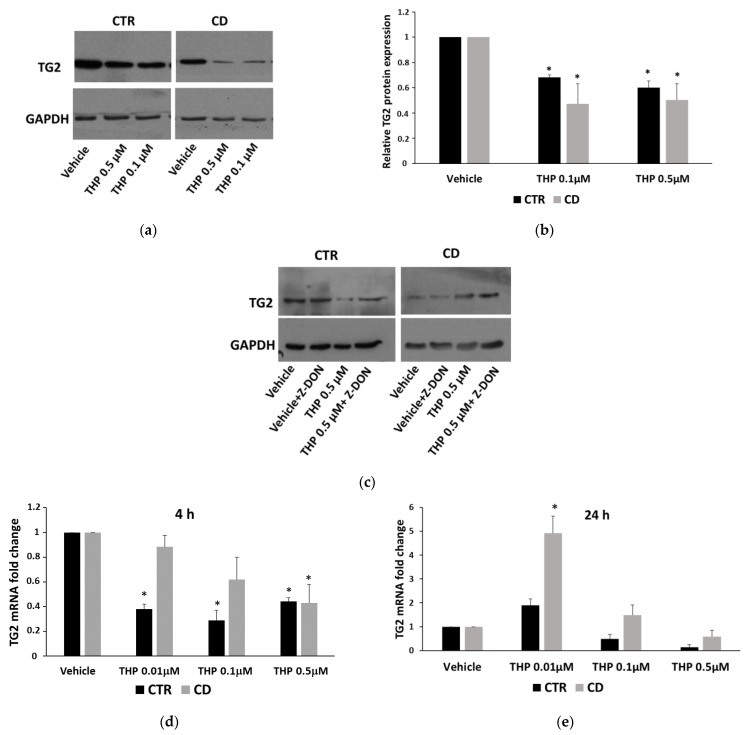
TG2 expression in the presence of THP. (**a**) Representative Western blot anti-TG2 on samples from one control and one celiac culture treated with THP for 24 h. (**b**) Densitometric analyses relative to Western blot anti-TG2 on samples from three control and three celiac cultures treated with THP for 24 h. (**c**) Western blot anti-TG2 on samples from one control and one celiac culture treated with THP for 4 h. (**d**,**e**) Relative TG2 mRNA levels measured in samples from three control and three celiac cultures treated with THP for 4 h and for 24 h, respectively. Protein and mRNA levels are normalized with respect to GAPDH expression. Data are reported as mean ± SE. * *p* < 0.05 vs. respective vehicle.

We verified whether the reduced Ca^2+^ ions release from ER of CD fibroblasts could imply a consequence in relation to the TG2 transamidating activity. We performed an in-situ TG assay, by using the substrate biotin-pentylamine, in cells treated for 30 min with THP at different concentrations. As shown in Figure 10, in non-CD cells, THP induced a dose-dependent increase of TG2 activity, whereas, in CD fibroblasts, we were not able to detect TG2 activation, at any THP concentration tested. We also evaluated TG2 expression after 24 h of treatment with THP. Western blot analysis indicated that THP caused a reduction of TG2 protein level in both CD and non-CD cells; such a reduction appeared slightly more pronounced in CD samples (Figure 11a,b). However, a Western blot analysis performed after 4 h of treatment showed that the TG2 level diminished only in control cells (Figure 11c). Moreover, the TG2-specific inhibitor Z-DON (40 µM) contrasted the diminution of TG2 induced by THP in control cells, whereas it didn’t have an evident effect in CD cells (Figure 11c). The analysis of TG2 transcripts showed that, after 4h of treatment, TG2 mRNA level was reduced (Figure 11d), whereas, after 24 h of treatments, a moderate increase of TG2 expression at the lowest concentration of THP in celiac cells was observed (Figure 11d).

**Figure 12:**
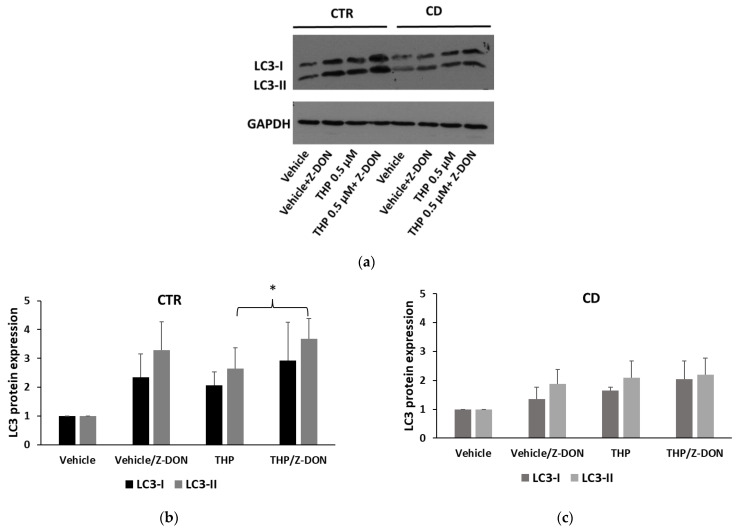
LC3 expression in the presence of the TG2 inhibitor Z-DON. (**a**) Representative Western blot anti-LC3 on samples from one control and one celiac culture in the presence of THP, Z-DON, and both for 24 h. (**b**,**c**) Densitometric analyses relative to Western blot anti-LC3 on samples from three control and three celiac cultures, respectively, in the presence of THP, Z-DON, and both. Protein levels of LC3 I and LC3-II are normalized with respect to GAPDH expression. Data are reported as mean ± SE. * *p* < 0.05 as indicated (Student’s *t*-test for unpaired data).

Based on the observation that TG2 activity has a role in favoring the autophagic flux [13], we tested the effect of Z-DON in modulating the level of LC3. In both control and CD fibroblasts, we observed that Z-DON increased basal levels of LC3-I and LC3-II (Figure 12), indicating a higher expression of LC3 protein and an accumulation of autophagosomes. When cells were treated with THP in the presence of Z-DON, we observed a further increase of LC3-II form, with respect to samples treated with THP, but only in control cells, not in CD ones (Figure 12). This observation could imply that, in control cells, the TG2 inhibitor was delaying autophagy, as expected, whereas, in CD cells, Z-DON was less effective, being autophagy in CD cells basally delayed.

## 3. Discussion

Increasing experimental evidence indicates that cells of CD patients display constitutive differences with respect to the cells of healthy subjects, which, on the whole, are able to confer to CD patients a greater susceptibility to be negatively affected by exposure to dietary gluten and other proinflammatory stimuli [14]. In particular, alterations of vesicular trafficking, cytoskeleton structure, signaling, and of proliferative and inflammatory pathways have been described in intestinal and non-intestinal cells from subjects on a gluten-free diet [15,16,17,18,19]. Recently, the Ca^2+^-dependent enzyme TG2 has been also considered an important player in the definition of the cellular celiac phenotype, a condition that is independent of gluten exposure and manifests also far from the gut, the primary site of inflammation [9,20,21]. Besides a differential TG2 subcellular distribution in celiac and control skin-derived primary fibroblasts, we previously described a different TG2 activation in the presence of P31-43 [9]. Since P31-43 is able to mobilize Ca^2+^ ions from both mitochondria and ER [12], one possible explanation of the differences we observed is that normal and CD cells could possess a different regulation of Ca^2+^ homeostasis.

In this work, we used a brief pulse of the sarco(endo)plasmic reticulum Ca^2+^ ATPase (SERCA) inhibitor THP as a tool to elicit rapid ER Ca^2+^ release and verify the endogenous ER Ca^2+^ level in both CD- and HLA DQ2/8 negative non-CD skin-derived fibroblasts. Used for a longer period, THP is an ER stress-inducer that, by inhibiting the SERCA pump, leads to an ER Ca^2+^-depletion, thus triggering the UPR aimed to restore ER homeostasis [22]. On the bases of the duration of exposition and of exposing concentration, THP could produce acute cytotoxic effects and cell death [23]. The performed cell-viability assay showed that CD cells were constitutively less sensitive to THP-induced cytotoxicity than non-CD ones. This different behavior could be related to a higher rate of cell proliferation in CD cells [15], despite the presence of the toxic agent. By performing the microfluorimetric analyses, we found a constitutively lower basal [Ca^2+^]i in CD fibroblasts than in control cells; we also registered a very low release of Ca^2+^ ions from ER in CD cells with respect to controls, suggesting that a lower amount of Ca^2+^ is stored in ER of CD cells than of control ones. Even if the literature reports studies about Ca^2+^ malabsorption at the intestinal level or about less Ca^2+^ excretion whit urines in CD patients [24,25], data on a different modulation of Ca^2+^ homeostasis in CD cells, and on the biological consequences of this, have not been reported.

The analysis of UPR in CD and non-CD cells revealed a condition of lower adaptability of CD cells to a stress agent. First, we found a lower basal level of GRP78 in CD cells than in controls; this finding could indicate that CD cells are less capable of rapidly adapting to an ER stress condition. Data on induction of GRP78 expression by THP, on the whole, suggest that UPR was more intense and prolonged in CD fibroblasts than in non-CD cells, as demonstrated by a higher expression of the GRP78 mRNA after 4 h of treatment with THP and by a marked production of the protein after 24 h at the higher THP concentration. Data on XBP1 splicing also support the idea that, in CD cells, UPR could be more intense and prolonged. In line with these observations, we also found that THP-stimulated CD fibroblasts produced a higher amount of mRNA for a late marker of ER stress, CHOP, than non-CD fibroblasts (mean mRNA fold increase of 13.7 for control samples and of 32.1 for CD samples, after treatments of 4 h with THP 0.5 μM).

Recent evidence suggests that prolonged or excessive ER stress induces not only cell death but could also amplify cytokine-mediated inflammatory response that is particularly relevant in some intestinal inflammatory diseases [26,27]. Moreover, sustained UPR signaling could activate autophagy to enhance the degradative capability of misfolded proteins and damaged ER. In this view, autophagy contributes to restoring cellular homeostasis and mitigating inflammation. Thus, we investigated how THP modulated autophagy in non-CD and CD fibroblasts, by analyzing two typical markers: LC3, in particular the LC3-II form, whose accumulation is indicative of the formation of autophagic compartments [28], and p62, whose decrease indicates its degradation during the progression of the autophagic flux [29]. Interestingly, we found that the basal ratio of LC3-II/I isoforms was higher in CD cells than in control samples and also that, in CD cells, there was a more consistent presence of perinuclear LC3-positive vesicles, suggesting a constitutively higher presence of autophagosome in CD cells than in control ones. Moreover, p62 basal levels were higher in CD cells than in non-CD ones, indicating a possible delay in the normal progression of the autophagosome. In the presence of THP, we observed that, in non-CD cells, LC3-II increased and p62 reduced. These findings indicated, as expected, the progressive maturation of the autophagosome in the presence of THP. In CD cells, we also found an increase of LC3-II, but the p62 level did not diminish and even increased, suggesting that the autophagic flux could be delayed or partially blocked. Experiments performed in the presence of a starvation medium and/or of bafilomycin A1, an autophagy inhibitor that causes the increase in lysosomal/vacuolar pH [30], support this idea. Indeed, in controlled fibroblasts, a normal autophagic flux occurs, as indicated by an increase of LC3-II in starved cells and in cells treated with bafilomycin A1, and a further increase in starved cells treated with bafilomycin A1. On the other hand, in CD cells, starvation was less effective in inducing the increase of LC3-II isoform, and treatment with bafilomycin A1 of non-starved and starved cells induced a similar increase of LC3-II.

Since the cytosolic Ca^2+^ level is considered a signal to activate autophagy [31], we hypothesize that a reduced [Ca^2+^]i and a low release of Ca^2+^ from ER could contribute to accumulate autophagosomes in CD cells treated with THP. Recently, it has been demonstrated that in Caco-2 cells gliadin peptides were able to impair autophagy [32,33]. Therefore, it is possible to speculate that gliadin peptides could exacerbate the CD phenotype regarding defective autophagy. 

In our experimental conditions, THP-induced Ca^2+^ release from ER appeared too low to allow the detection of TG2 transamidating activity in CD cells. This could explain why we previously found a low TG2 activation after stimulation with P31-43 [9]. In that case, TG2 was probably activated mainly by Ca^2+^ ions released from mitochondria. A low TG2 activation in CD cells could represent another factor that contributes to a negative modulation of autophagy; indeed, it has been reported that transamidating activity is required for the proper completion of autophagic flux [13]. TG2 is also degraded into autophagosomes during their maturation [13,34]. In CD cells treated for 4 h with THP, TG2 reduction was less evident than in control cells, probably due to a reduced degradation into autophagosomes. In line with the role of TG2 activity in the autophagic flux, the TG2 inhibitor Z-DON clearly reduced TG2 degradation and caused an increase of LC3 expression in non-CD cells. These effects were less evident in CD fibroblasts, suggesting that in these cells, constitutive delayed autophagy possibly occurred. An impaired autophagy could explain why TG2 and LC3 colocalize more in CD fibroblasts than in controlled ones [9]. A little increase of TG2 mRNA, in CD cells only, could contribute to refurnishing the TG2 protein pool, as a tentative to allow the maturation of autophagic vesicles.

In conclusion, we used a model of skin-derived fibroblasts to demonstrate constitutive differences between normal and celiac cells. The use of this cellular model offers the opportunity to study molecular features of celiac cells regardless of gluten exposure and in a site far from the primary site of inflammation, i.e., the intestinal mucosa. We demonstrated that, in CD cells, Ca^2+^ homeostasis was constitutively altered, thus causing both a more intense UPR and, probably, an engulfed autophagy in response to stimulation with THP, also related to a lower TG2 activity. Altogether, our findings, in line with previously reported ones [14], indicate that CD cells could be less capable of responding to stressor agents in a physiological way and adapting to a stress condition. Of course, it would be interesting to confirm the peculiar response to THP also in cells from the intestinal district. However, with our work, we have added a further piece of knowledge on the complex CD pathogenesis and on mechanisms at the bases of the constitutive vulnerability of CD cells.

## 4. Materials and Methods

### 4.1. Skin-Derived Fibroblast Cultures and Treatments

Fibroblasts were obtained from skin biopsies of five CD patients on a gluten-free diet (age range 17–43) and five HLA-DQ2/8-negative healthy subjects (age range 25–30 years). CD patients were on a gluten-free diet for at least 4 years and showed normal (Marsh T_0_) biopsies, negative serology, and negativity for anti-endomysium antibodies. None of the CD patients were affected by dermatitis herpetiformis. Cells were cultured in Dulbecco’s Modified Eagle’s Medium supplemented with 20% (*v*/*v*) fetal bovine serum, 1 mM L-glutamine, 50 U/mL penicillin, and 50 µg/mL streptomycin (Invitrogen SRL, Milan, Italy). Cells were maintained at 37 °C in a 5% CO_2_, 95% air-humidified atmosphere. All experiments were performed on fibroblasts at passages between 4 and 7. For cell treatments with THP or bafilomycin A1 (Merck, Milan, Italy), cells were seeded in six-well plates and grown until they reached the confluence of 70–80%. THP was dissolved in DMSO at 1 mM, then conserved at −20 °C in small aliquots. Working dilutions were made in DMSO; cells were treated at THP concentrations ranging from 0.01 µM to 1 µM for indicated times. Bafilomycin A1 was purchased as a ready-to-use solution in DMSO (0.16 mM) and was used at 1 µM for 4 h. Treatments with bafilomycin A1 were preceded by starvation for 2 h in a medium without amino acids, supplemented with 0.1% (*v*/*v*) fetal bovine serum and 1% (*w*/*v*) bovine serum albumin.

### 4.2. Cell Viability Assay

Skin-derived fibroblasts were seeded in 96-well plates at 3.5 × 10^3^ cells/cm^2^, cultured for 48 h, and treated with different amounts of THP for further 24, 48, or 72 h. Cell viability assay was performed by employing the reagent 3-(4,5-dimethylthiazol-2-yl)-2,5-diphenyltetrazolium bromide (MTT) (Merk), added at 0.5 mg/mL to the cell medium and incubated for 90 min at 37 °C. The resulting formazan crystals were dissolved in DMSO, then samples were analyzed by a microplate reader, measuring absorbances at 595 nm (deducing the background signals at 655 nm). Cell viability was expressed as % of viability registered in untreated cells. For each cell culture, two independent experiments, each in triplicate, were performed.

### 4.3. Ca^2+^ Concentration Measurements

Intracellular Ca^2+^ concentration ([Ca^2+^]i) was measured by single-cell computer-assisted video imaging using the Ca^2+^ indicator Fura-2AM. Briefly, fibroblasts were seeded on glass coverslips coated with 30 μg/mL poly-L-lysine (Merk). Then, they were loaded with 10 μM of Fura-2AM for 30 min at 37 °C in a normal Krebs solution consisting of 5.5 mM KCl, 160 mM NaCl, 1.2 mM MgCl_2_, 1.5 mM CaCl_2_, 10 mM glucose, and 10 mM Hepes-NaOH, pH 7.4. At the end of the loading period, the coverslips plated with fibroblasts were perfused in a perfusion chamber (Medical System, Greenvale, NY, USA) mounted onto an Axiovert200 microscope (Carl Zeiss, MicroImaging Inc., Iena, Germany) equipped with a FLUAR 40× oil objective lens, a digital imaging system composed of a MicroMax 512BFT cooled CCD camera (Princeton Instruments, Trenton, NJ, USA), LAMBDA10–2 filter wheeler (Sutter Instruments, Novato, CA, USA), and Meta Morph/MetaFluor Imaging System software (Universal Imaging, New York, NY, USA). Fura-2AM fluorescence intensity was measured every 3 s. Ratiometric values were automatically converted by the software to [Ca^2+^]i [35,36]. To selectively deplete the ER Ca^2+^ store, experiments were performed in the presence of THP (1 µM).

### 4.4. TG2 Assay

TG2 activity was monitored by an in-situ assay as previously described [9]. For each cell culture, at least two independent experiments, each in triplicate, were performed. Briefly, fibroblasts were treated for 30 min at 37 °C with THP at concentrations 0.01, 0.1, and 0.5 µM, in the presence of the TG2 substrate pentylamine-biotin (Thermo Fisher Scientific, Monza, Italy) (0.5 mM); in some experiments, the TG2 inhibitor Z-DON (Zedira, Darmstadt, Germany) was employed at 40 µM. Then, cells were harvested in RIPA lysis buffer (20 mM Tris pH 7.5, 150 mM NaCl, 10% glycerol, 0.1% SDS, 1 mM Na_3_OV_4_, 1 mM PMSF, and a cocktail of protease inhibitors). After lysis, proteins (25 µg) were coated overnight in wells of a 96-well microplate, incubated with a blocking solution (10% bovine albumin solution in borate buffer saline), then with horseradish peroxidase-conjugated streptavidin (Thermo Fisher Scientific) 1:3000. Finally, peroxidase enzymatic reaction was performed by adding 3,3′,5,5′-tetramethylbenzidine. Absorbances measured at 450 nm were used as an estimation of the intracellular TG2 activity.

### 4.5. Western Blot

Western blot analyses were performed to detect the expression of specific proteins. Fibroblasts were treated with THP at different concentrations for the specified times, then lysed in RIPA buffer. For each cell culture, at least two independent experiments were performed. Proteins (45–60 µg) of total lysates were loaded onto denaturant polyacrylamide gels, then electro-transferred on polyvinylidene difluoride blots. A blocking steps was performed by using a 5% skim milk solution; then blots were incubated overnight at 4 °C in a 1:1000 dilution in 0.5% buffered skim milk of the following primary antibody: mouse anti-GRP78, mouse anti-TG2 (clone CUB 7402) (Thermo Fisher Scientific), rabbit anti-LC3II antibody, mouse anti-p62 antibody, and mouse anti-GAPDH antibody (Santa Cruz Biotechnology, Milan, Italy). As secondary antibodies, horseradish peroxidase-conjugated anti-mouse or anti-rabbit (Bio-Rad Laboratories, Milan, Italy) antibodies were used for 1 h; finally, immunocomplexes were revealed using a chemiluminescence detection kit (Merck) according to the manufacturer’s instructions.

### 4.6. PCR

To quantify TG2, GRP78 and CHOP mRNA levels after treatments with THP for 4 h and 24 h, real-time PCR analyses were performed according to protocols previously reported [20,37,38]. For each cell culture, at least two independent experiments were performed. To evaluate the unspliced and the spliced form for the XBP1 transcript, after treatments with THP for 4 h, a conventional RT-PCR was performed as previously reported [37,38,39], followed by the visualization of the obtained cDNA in a 2.5% agarose gel stained with ethidium bromide.

### 4.7. Confocal Immunofluorescence

To visualize LC3 in fibroblasts, a confocal microscopy approach was employed. Fibroblasts were grown on glass coverslips and treated with starvation medium and/or bafilomycin A1 for the indicated times. Then cells were fixed for 10 min with 3% paraformaldehyde and permeabilized for 5 min with 0.2% Triton-X 100. Cells were incubated for 1 h with the anti-LC3 rabbit antibody (1:100, in 1% bovine albumin serum), then with an anti-rabbit tetramethylrhodamine-conjugated antibody (1:100, in 0.1% bovine serum albumin) (Thermo Fisher Scientific). After washing, coverslips were mounted with Mowiol (Merck), and images were acquired with a Zeiss LSM 510 laser-scanning microscope (Carl Zeiss MicroImaging Inc.). The magnification of the micrographs was the same for all the figures shown (63× objective).

### 4.8. Statistics

Statistical analysis was performed using Student’s *t*-test for paired data. When the test for unpaired data was used, it was directly specified in the legend of the figure. Differences were considered to be statistically significant at *p* < 0.05.

## Data Availability

Data not presented in this manuscript are available on request from the corresponding author.

## Abbreviations

CD	celiac disease
ER	endoplasmic reticulum
Fura-2AM	Fura-2 acetoxymethyl ester
GRP78	78-kDa glucose-regulated protein
HLA MTT	human leukocyte antigens 3-(4,5-dimethylthiazol-2-yl)-2,5-diphenyltetrazolium bromide
P31-43	α-gliadin peptide 31-43
SE	standard error
TG2	type 2 transglutaminase
THP	thapsigargin
UPR	unfolded protein response
XBP1	X-box binding protein 1
